# Gottfried Wilhelm Osann (1833, 1836) on Simultaneous Color Contrast: Translation and Commentary

**DOI:** 10.1177/2041669517717755

**Published:** 2017-07-05

**Authors:** Robert P. O’Shea, Urte Roeber, Nicholas J. Wade

**Affiliations:** Discipline of Psychology, School of Psychology and Exercise Science, Murdoch University, Perth, Australia; Discipline of Psychology, School of Health and Human Sciences, Southern Cross University, Coffs Harbour, Australia; Discipline of Psychology, School of Psychology and Exercise Science, Murdoch University, Perth, Australia; School of Psychology, University of Dundee, UK

**Keywords:** color perception, simultaneous color contrast, optical superimposition, tachistoscope, methods for research in visual perception, Osann, history

## Abstract

Gottfried Wilhelm Osann (1796–1866) was a German scientist most renowned for his work in chemistry and physics. However, inspired by Goethe’s work on color, he published a paper on simultaneous color contrast in 1833 using a method that is similar to that of later authors: reflection of an achromatic spot from an angled piece of glass. He wrote at least four more papers on color contrasts, in 1836 using essentially the same method as credited to others. We provide a description and translation of Osann’s 1833 paper and the relevant part of his 1836 paper, say why these papers are interesting and important, give some biographical information about Osann, comment on the fate of Osann’s papers, and describe Osann’s other papers on color.

## Introduction

**Figure 1.**
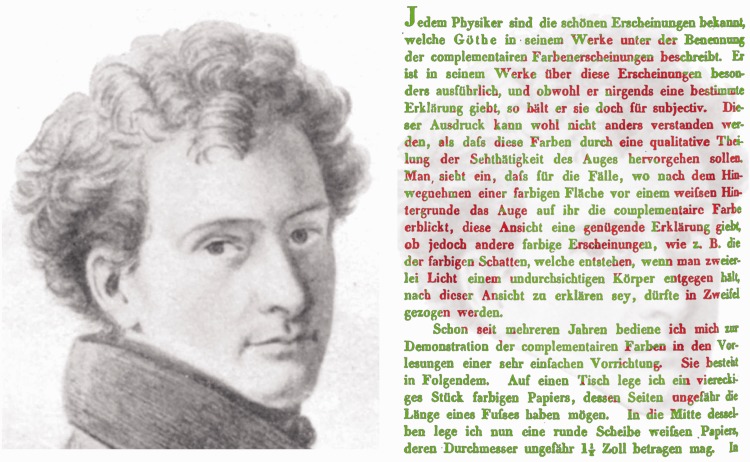
Left. Detail of a portrait of Gottfried Wilhelm Osann (1796–1866) derived from a drawing that belonged to Adele Schopenhauer. (The portrait is in the public domain: https://commons.wikimedia.org/wiki/Category:Gottfried_Osann#/media/File:Gottfried_Osann_1823.png). Right, *Osann’s complementary colours* by Nicholas Wade. Osann’s portrait can be seen in the text from the first page of his article on contrast colors (Osann, 1833).

Gottfried Osann (Figure 1, left) is best known for his part in the discovery of the element ruthenium (Hödrejärv, 2004). However, he retained a life-long interest in color phenomena, stimulated by his personal contacts with Goethe (Rinecker, 1867). This is evident from the text on the first page of Osann (1833), his first article on color contrast (Figure 1, right; see later for the translation).

Osann (1833) set forth a method to demonstrate simultaneous color contrast: the alteration of color in one region by surrounding color (e.g., Chevreul, 1839; Kirschmann, 1892; Smithson, 2015). This phenomenon is also referred to as contrast color (e.g., Helmholtz, 1867, p. 415; Whittle, 2003). Osann published his method and observation 14 years before the person usually cited (e.g., by Helmholtz, 1867, p. 405) for first publishing a similar method and the same observation: Ragona-Scinà (1847) (see O’Shea, Brini, & Wade, 2016).

**Figure 2.**
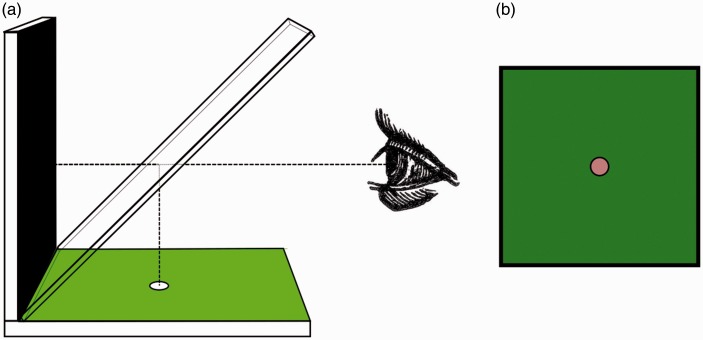
(a) Schematic representation of Osann’s (1833) apparatus. (b) How the display appears.

Osann’s (1833) apparatus comprised an angled sheet of glass through which a black card was viewed directly and from which a white disk on colored cardboard was viewed by reflection. The reflected spot appears to have the color complementary to its background. Figure 2 shows a schematic representation for a green background.

Ragona-Scinà’s (1847) apparatus was slightly different: He used colored glass reflecting a black spot on white cardboard onto a white card. A useful movie of Ragona-Scinà’s apparatus and of an improvement made by Hering (1887) has been provided by Kalkofen (1993).

Three years after Osann’s first paper, he (1836) described an apparatus essentially the same as that of Ragona-Scinà (1847), using colored glass. Here is a translation of that part of the paper:

3) We put the piece of white paper in the middle of the black, and set instead of unstained glass a colored [glass] just on its right-hand edge. Now, if one looks from left to the right, the eye perceives the reflection of white paper stained with the complementary color of the glass. If the glass was green, one sees a red reflection, and so on. (Osann, 1836, p. 295)

Yet, as we will discuss later, Ragona-Scinà was accorded most of the credit for inventing a method to demonstrate simultaneous color contrast, whereas Osann was largely ignored, perhaps because Osann insisted in all relevant papers that the colors he saw were objective rather than subjective.

## Why Are Osann’s (1833, 1836) Papers Interesting and Important?

There are at least three reasons Osann’s (1833, 1836) papers are interesting and important:

1. They give one of the first, easy demonstrations of the creation of color in an achromatic region by a surround color, although similar phenomena had been known since antiquity (Wade, 1998). Aristotle commented on color contrasts in dyed fabrics: “In woven and embroidered stuffs the appearance of colours is profoundly affected by their juxtaposition with one another (purple, for instance, appears different on white and on black wool), and also by differences of illumination” (Ross, 1931, p. 375a). Ibn al-Haytham (Sabra, 1011–1021/1989) gave directions on how to see contrast effects with paints. He appreciated that the perception of colors was always comparative. The example he gave was of green paint on different colored backgrounds, and it was painters who recognized the significance of color contrasts. Experimental investigations have their origins in the late 18th century. Monge (1789) (see translation by Kuehni, 1997) reported that a bright, white spot, from sunlight projected through a colored, translucent material such as taffeta, appeared to have the complementary color. Mollon (2006) noted that this paper faded into obscurity. Young (1807/1971) illustrated the influence of surrounding a gray patch with different colors, as did Goethe (1810, 1840/1970). Brandes (1827) had achieved something similar to Osann’s (1833) demonstration using reflection of a window from the front and rear surfaces of a sheet of colored glass. We have tried this, and it is very difficult to see the reflections and that one has the complementary color of the glass.
2. They were the first use in vision research of using angled glass to superimpose images of two different objects, anticipating its use in multifield tachistoscopes, as O’Shea et al. (2016) have argued.
3. They potentially provided a challenge to an extant, influential theory that color vision arises from some property of light we now call wavelength. This was proposed by Young (1804) who extended Newton’s (1704) theory. Although Young was aware of various kinds of color contrast phenomena (Mollon, 2006), his explanation along the lines of Monge’s (1789) put these into the realm of subjective phenomena as Brandes (1827) made clear by calling them that. There is no preponderance of any particular wavelength in Osann’s (1833, 1836) achromatic spots as we now know.

## Who Was Osann?

Gottfried Wilhelm Osann (Figure 1) was born in Weimar on October 26, 1796 and died in Würzburg on August 10, 1866 (see Rinecker, 1867 for details of his life). He was the youngest of five children. When Osann was nine, his father died. His mother remarried Friedrich Heinrich Gotthelf, a member of the Weimar literary circle that included Johann Wolfgang Goethe and Adele Schopenhauer (sister of philosopher Arthur Schopenhauer; she became Osann’s close companion from 1820 to 1826). It is likely that Osann’s discussions with Goethe, his acquaintance with Goethe’s work on color (e.g., Goethe, 1810, 1840/1970), his possible acquaintance with Arthur Schopenhauer’s work on vision and colors (Schopenhauer, 1816), and his stepfather’s urging all combined to influenced him to study science. Two of Osann’s brothers preceded him into academia: Emil, a professor of medicine who founded the science of treating disease by bathing, and Friedrich, who was a professor of philology.

During his career, Osann first devoted himself to chemistry, publishing several acclaimed books. His interests in color were aroused in his youth: “His researches on color phenomena clearly originated from his time in Weimar and were stimulated by Goethe” (Rinecker, 1867, p. XLIX). This resulted in publications on color from 1833 to 1866 yielding at least five papers on color contrasts (Osann, 1833, 1836, 1837, 1847, 1860).

Osann began lecturing in physics and chemistry in 1819 at University of Erlangen. He moved to University of Jena in 1821, then back to University of Erlangen in 1823. He was appointed Professor of Chemistry and Pharmacy at University of Dorpat (now University of Tartu) in 1823, then Professor of Physics and Chemistry at University of Würzburg in 1828—a position he held until his death. He is best known for his work at Dorpat on samples of platinum ore from the Ural mountains; he extracted what he considered to be a new element which he named ruthenium (Osann, 1826, 1828). As Hödrejärv (2004) noted, Osann’s part in the discovery of ruthenium is often, coincidentally, overlooked.

## What Was the Fate of Osann’s (1833, 1836) Papers?

Ragona-Scinà’s (1847) paper was accorded most of the credit for using angled glass to show simultaneous color contrast. O’Shea et al. (2016) found 23 citations of Ragona-Scinà’s (1847) paper, including one in the 21st century. We have been able to find only eight mentions of Osann’s (1833) work on color contrast and only two, Fechner (1838) and König’s bibliography for (Helmholtz, 1896), correctly cited and referenced the paper. We have been able to find only a few citations of Osann (1836), including by Plateau (1878).

We propose five reasons Osann’s (1833, 1836) papers have drifted into obscurity:

1. Whereas Helmholtz (1867) credited Ragona-Scinà (1847) with introducing the mirror method and gave a (misleading) diagram of his apparatus (p. 405, see also Helmholtz, 1924, p. 283), he cited Osann (1833, 1836, 1837) only briefly for proposing that colored shadows are objective and for using colored glass to show contrast colors.
2. Osann (1833) made a mistake by arguing that the colors he saw were objective. He concluded this after he reported seeing the same colors when he looked at the reflected spot through a small hole that blocked the reflected view of the colored surround.^1^ In 1836, Osann proposed that the colors in the reflection of the achromatic region arose from some optical properties of the reflected light. Yet when Fechner (1838) repeated Ossan’s observation through a hole that blocked the view of the colored surround, he saw the reflected spot as achromatic. Fechner adduced other arguments and new demonstrations to show that the color created in an achromatic region is subjective. Fechner attributed Osann’s experience to color memory—the tendency to see a stimulus as having its remembered color (e.g., Collins, 1932).^2^ Nevertheless, in his last word on the matter, Osann (1860) reiterated his argument that the colors are objective.
3. Ragona-Scinà (1847) did not cite Osann (1833) or (1836).
4. Three authors who did name Osann as originating the mirror method for showing simultaneous color contrast—Oliver (1885, p. 255), Kalkofen (1993), and Turner (2014, p. 110)—did not give any reference. Indeed, Oliver misspelled Osann’s name as Ossau.
5. The only other author who did cite Osann (1833)—Zagaru'lko (1948)—cited him not for simultaneous color contrast, but incorrectly for the color of afterimages.

## What Were Osann’s Other Papers on Color?

In 1836, Osann considered afterimages and colored shadows, and how these various phenomena could be explained by the physics of light interacting with the physiology of the eye.

In 1837, Osann considered afterimages and colored shadows again. He considered colored shadows of an object from a candle flame and skylight—the shadows are blue and yellow, respectively—and argued that they are objective (indeed Mollon, 2006, showed that the former contained predominantly short-wavelength light and the latter predominantly long-wavelength light). Osann apparently missed the paper by Count Rumford (Thompson, 1794) showing that the blue color remains even when he used identical white-light sources, one through a yellow filter.

In 1847, Osann reported observations consistent with skylight’s being blue.

In 1860, Osann maintained his various arguments, visiting divers topics including dark adaptation, acoustics, bioluminescence, phosphorescence, polarization colors, and colors of lakes. He also described apparatuses for easily seeing afterimages (a tachistoscope), for allegedly demonstrating the objective nature of contrast colors, and for demonstrating the objective nature of colored shadows.

In the year of his death, Osann reported on the lines visible in the prismatic spectrum (Osann, 1866). Thus issues of color vision were an abiding source of experimental interest for most of his scientific life.

## Translation of Osann (1833)

*XVIII. Description of a simple apparatus to elicit so-called complementary colors, and evidence that the colors elicited with it are objective;*

*by G. [H.] Osann.*

Every physicist knows of the beautiful occurrences that Goethe calls complementary color phenomena. In his work, he especially elaborates on these phenomena, and even though he does not give a specific explanation at any point, he deemed them subjective. This expression cannot be understood other than that these colors result from a qualitatively different visual activity of the eye [Sehtätigkeit des Auges] from that supporting objective colors. One appreciates that this view offers a sufficient explanation for when the eye beholds the complementary color on a white background after a colored area (one has looked at) is removed from in front of it; however, it could be doubted whether it explains other color phenomena, for example, colored shadows that arise from shining two sorts of light on an opaque object.

For several years, I have used a very simple apparatus to demonstrate complementary colors in my lectures. It consists of the following. On a table, I put a square piece of colored paper, the sides of which may be approximately one foot. In its center, I now put a disc of white paper, the diameter of which may be approximately 1½ inches. At some distance behind the table, which I estimate to be four feet, a sheet of black paper is attached. Now I tilt a glass plate toward the colored paper such that it forms an acute [offenen] angle toward me. Next I place myself in front of the glass panel such that the eye receives the reflection of the white disc from the reflecting glass plate. The eye then beholds on the black paper behind the glass panel the disc colored with the color that is complementary to that of the colored paper. The coloring of the disc changes of course with the modification of differently colored papers one puts underneath the disc.

**Figure 3.**
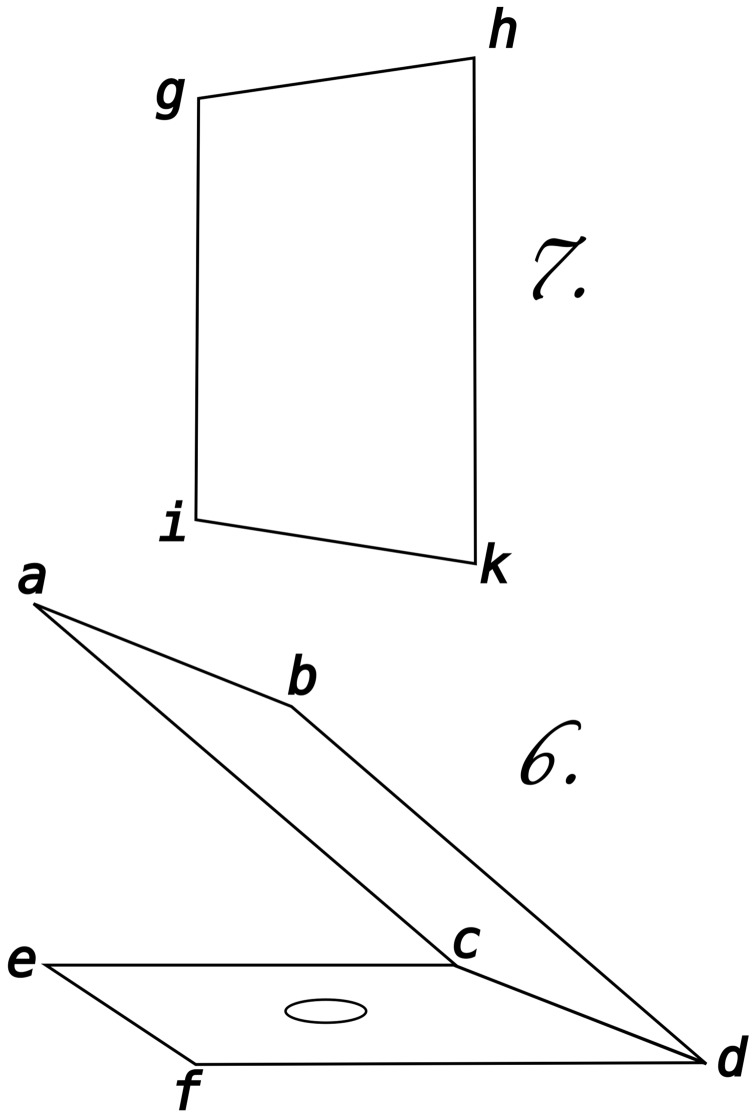
Description of the apparatus. *a b c d*, Plate V Figure 6, is the glass plate; for which ordinary window glass can be used. *e c d f* is the colored paper in the center of which the white paper disc is put. *g h i k*, Plate V Figure 7, is a sheet of black paper in the center of which the eye sees the disc colored in complementary color. *a b e f* is the piece of cardboard (through which one may view the reflection of the disk without any reflection of the colored paper).

Now one should get a square piece of cardboard of the same size as the colored paper; in its center one should make a circular opening of a slightly smaller diameter than the white disc and one should place it vertically in front of the angle that the glass plate makes with the colored paper (Figure 6 Plate V; Figure 3). If one now looks through the opening by putting the eye closely in front of it, one perceives the aforementioned phenomenon. Doing it this way does not decide on the question whether the phenomenon is of subjective or objective nature, but it will be decided as soon as one changes the experiment as follows. One steps back a little while keeping an eye on the opening. Relatively to the distance of stepping back, the reflection of the coloring of the surround (that results from the reflection of the colored paper) decreases, and soon one reaches the distance at which the rim of the disc coincides with the rim of the opening.

Now the eye does not perceive anything of the reflection of the colored paper, notwithstanding this, one still sees the disc colored with the complementary color as before, in fact without the slightest attenuation. The same occurs if one starts looking through the opening from this distance right away, when no reflection of the colored paper at all gets into the eye.

Everybody understands that, if this phenomenon were caused by a qualitatively different visual activity of the eye, the coloring of the disc would attenuate by the amount that one moves the eye away from the opening, and that finally in the moment of complete disappearance of the reflection of the colored paper the complementary color of the disc should also disappear. This does not happen, and that it does not happen proves most decisively that this phenomenon is not of subjective but of objective nature.

Furthermore this experiment shows that the phenomenon is a catoptric phenomenon, because the reflection of the disc reaches the eye in the same angle in which the light reaches the glass disc, of which one can easily convince oneself by tilting the disc toward the colored paper.

Würzburg March 21, 1833.

## Conclusion

We have shown that Osann (1833, 1836) made significant contributions to research in visual perception by pioneering the “mirror contrast” (Oliver, 1885, p. 255) method or “Ragona Seinà” (Breese, 1899, p. 44) method that became popular with others both to show simultaneous color contrast and to research all sorts of phenomena until optical methods of superimposing images were largely supplanted by computer monitors. We hope our paper goes some way to restoring Osann’s primacy with the method, if not his primacy in demonstrating easily how an achromatic region can appear colored from simultaneous color contrast.

## Notes

1 No less of a color scientist than W. A. H. Rushton FRS (1901–1980) reported a similar observation (Rushton, 1961) when viewing Land’s display of a scene made by optically superimposing an achromatic photograph of the scene taken through a red filter and projected through a red filter onto an achromatic photograph of the scene taken through a green filter (Land, 1959). When Rushton looked at an area of the display that appeared green, then looked at the same area through a reduction tube, he said: I looked at the “green” region through a tube narrow enough to cut out all the surround. Interposing the tube or removing it made no difference at all to the “green” color. (Rushton, 1972, p. P29). Rushton had the advantage over Osann of knowing the chromaticity of the area in question (pink), yet he too could not immediately shake the memory of having seen the area as green.

2 Color memory (e.g., Collins, 1932) is different from memory color—the tendency to see an achromatic version of an object, such as a banana, as having its typical color (Hering, 1874/1964; Witzel & Gegenfurtner, 2014).
